# Study of the behavior of snails intermediate hosts of *Schistosoma spp.* under different maintenance conditions and their resistance to salinity in an african laboratory environment

**DOI:** 10.1016/j.heliyon.2022.e10289

**Published:** 2022-08-19

**Authors:** Fatou Thiam, Cheikh Bintou Fall, Papa M. Gaye, Bruno Senghor, Arfang Diamanka, Amélé N. Wotodjo, Kokou Abotsi, Philippe Parola, Babacar Faye, Cheikh Sokhna, Doudou Sow, Souleymane Doucouré

**Affiliations:** a VITROME, IRD-UCAD International Campus of the Institute of Research for Development, Dakar, Senegal; b Department of Animal Biology, Faculty of Sciences and Techniques, University Cheikh Anta Diop of Dakar, Senegal; c Department of Parasitology-Mycology, Faculty of Medicine, Pharmacy and Odontology, University Cheikh Anta Diop of Dakar, Senegal; d Aix-Marseille University, IRD, AP-HM, SSA, VITROME, Marseille, France; e Institut Hospital-University (IHU)-Mediterranean Infection, Marseille, France; f Department of Parasitology-Mycology, UFR Health Sciences, University Gaston Berger, Saint-Louis, Senegal

**Keywords:** Snail, Schistosomiasis, Water, Salinity, Survivor, Size

## Abstract

**Background:**

The control of snails intermediate hosts remains an effective strategy to limit schistosomiasis transmission despite the widespread mass de-worming campaign based on praziquantel. Therefore, the study of snail biology could help to improve snails control strategies. This study evaluated the development of *Biomphalaria pfeifferi*, *Bulinus senegalensis* and *Bulinus truncatus* in various water sources and their resistance to salinity.

**Methods:**

Five day-old juveniles individuals issued from adult snails non-shedding *Schistosoma spp* cercariae were breed in distilled water, commercial mineral water, ground pump water and well water. Snail’s survival rate and size were measured over a period of 40 days. These two parameters were also measured over 30 days in increasing saline solutions (1 g/l, 3.5 g/l and 4 g/l) made of well water to which sodium chloride was added.

**Results:**

*B. truncatus* growth was not hampered by any water sources with a survival rate between 68% and 84% (log rank X^2^ = 1.86, df = 3, p = 0.60). Despite a poor survival rate (8%) in distilled water, *B. pfeifferi*, has adapted to other water sources with a survival fraction between 88% and 96% (log rank X^2^ = 61.94, df = 3, p < 0.0001). *B. senegalensis* development was very delicate with low survival rate of 4% in distilled water, 20% in well water and 24% in commercial mineral water and ground pump water (log rank X^2^ = 13.24, df = 3, p = 0,004). For each species, even if the difference is not significant, the size of snails is larger with well water and pump water compared to distilled and commercial mineral water.

*B. pfeifferi* survival rate was at 45% in both three saline solutions at day 30. *B. senegalensis* population collapsed at day 10 in 4 g/l saline solution and persisted until day 30 in both 1.5 g/l and 3.5 g/l solution. *B. truncatus* also persisted with a survival rate at 20% in 1.5 g/l but collapsed at day 5 and 15 in 4 g/l and 3.5 g/l solution, respectively.

**Conclusion:**

The differences in adaptation between snails species show the need to take into account the water sources for snail breeding in the laboratory. Further studies could help to determine the optimal water quality for each snail species in order to standardize breeding conditions. This study could contribute to the understanding of the dynamics and distribution of snails in natural conditions.

## Highlights

• The knowledge of snail breeding conditions could represent a breakthrough for schistosomiasis control
• The survival rate of *B. truncatus*, *B. senegalensis* and *B. pfeifferi* depended on the origin of the water used for snails breeding
• Only *B. truncatus* displayed relatively high survival rate to both distilled water, commercial mineral water, ground pump water and well water
• The survival of *B. pfeifferi* is less hampered in saline water compared to *B. truncatus* and *B. senegalensis*

## Graphical abstract

Snails field collection, laboratory rearing and size measurement system.

**Figure:**
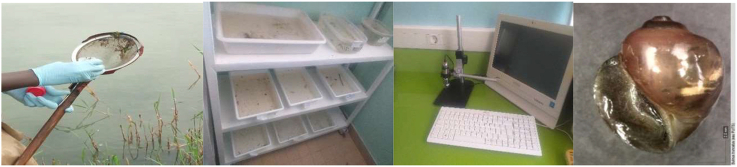

## 1 Introduction

Schistosomiasis represents a serious health issue in tropical regions and particularly in Sub-Saharan Africa (SSA). Urinary and intestinal schistosomiasis, the two forms of the disease are responsible of more than 200 million cases worldwide with 90% of cases occurring in SSA (WHO, 2020). Praziquantel (PZQ) drug is commonly used to treat the disease in endemics areas (Doenhoff et al., 2008). PZQ mass administration target most often the school aged children that represent the population at risk and the most important human reservoir of *Schistosoma haematobium* and *S. mansoni* parasites causing the disease (WHO, 2013, 2006). Poor socio-environmental conditions are most often the causes of the failures observed with the use of the PZQ mass administration (King, 2010). Indeed, in most endemic rural areas in SSA, the absence of running water is the cause of the use of ponds where human populations are in permanent contact with the snails of genus *Bulinus* and *Biomphalaria*, the intermediate hosts of the schistosomes parasites (Brown, 2005). This situation inevitably leads to human population re-infection and undermines the efforts to eliminate schistosomiasis (King et al., 2020). On the other hand, effective control of snail populations could effectively break the transmission cycle and ensure sustainable control of the disease (Sokolow et al., 2016). Before the widespread use of PZQ, the control of snail populations was the main strategy used to limit the disease transmission within human population and it is still considered as the key for schistosomiasis control (Shiff, 2017; Sokolow et al., 2016, 2018). However, the control of snail through the use of molluscicides or environmental management is very demanding and requires a good knowledge of the bio-ecology of snails (King et al., 2015; Odongo-Aginya et al., 2008). In the transmission areas, different species of snails vectors are sympatric with a very complex dynamic of their populations (Diakité et al., 2017; Senghor et al., 2015). The knowledge of their bio-ecology could help to improve the existing snail control strategies and to understand the role of different snail species on the schistosoma transmission and especially the emergence of hybrid strains (Haggerty et al., 2020). This certainly requires to carry out studies in the field, but also to set up experimental models to facilitate the study of snail behavior. The experimental model could help to better define the maintenance conditions of the snails in the laboratory and study their interaction with parasites. But also to study their adaptation to ecological changes that may impact their seasonal dynamics and spatial distribution. Natural phenomena and human activities can caused enormous environmental changes with significant impact (King et al., 2015) on the life history traits of snails and their potential to transmit *Schistosoma* parasites. For instance, the construction of dams on the Senegal River Basin (SRB) has created a more stable flow of water with reduced salinity favorable to *Biomphalaria pfeifferi* and *Bulinus globosus* leading to the emergence of schistosomiasis outbreaks in human and livestock (Southgate, 1997). Thus, to set up an experimental model, it is essential to have a malacological facilities, especially in endemic areas, to breed and study snails vectors under standardized conditions. In most of snails breeding protocol, the physico-chemical conditions such as temperature, humidity, pH, dissolved oxygen and the quality of the food and the water are the essential elements taken into account. Sometimes, natural water from snail breeding site or tap water is used during experimental procedures (Christensen et al., 1979; Kalinda et al., 2017; Watson and Al-Ali, 1961). However, this practice could induce biases during laboratory experiments due to the fact that the physico-chemical quality of these waters may change according environmental conditions or water treatment by chemical compounds. Also, the origin or the quality of the water is known to influence the results of experiments (Christensen et al., 1979; Donnelly et al., 1984). Thus, it is necessary to have a standard water quality in order to avoid sources of bias and lack of reproducibility in experimental protocols studying the snails. This will also help to determine the type of water which is sweetest for each snail species breeds in laboratory. In this study, we described the implementation of a malacological platform with a main focus to study the survival of *Biomphalaria pfeifferi*, *Bulinus truncatus* and *Bulinus senegalensis* reared indifferent water sources and their adaptation to salinity.

## 2 Materials and methods

### 2.1 Snail’s collections and morphological identification

The snails were collected during the year 2018 in Niakhar and Richard Toll, respectively in the Center-West and North in Senegal. In Niakhar, the transmission of *S. haematobium* occurs seasonally during the rainy period. The main snails species involved in *S. haematobium* transmission are *B. senegalensis* and *B. umbilicatus*. In Niakhar area, the ponds are temporary and depend on the intensity of the rainfall (Senghor et al., 2015), therefore, the collection of *B. senegalensis* specimens was made in October corresponding to the end of the wet season. In the North, in the SRB the transmission of both *S. haematobium* and *S. mansoni* are occurring permanently. The snails habitats are permanent (Bakhoum et al., 2019; Ndione et al., 2018). In this area, *B. pfeifferi* and *B. truncatus* were collected in July during the beginning of the rainy season. Scoop net was used to collect snail specimens that were placed in plastic container filled with few aquatic plants and water from the site of collection to keep them alive during their transfert to the laboratory. Then each specimen was morphologically identified using a key for snail identification (Brown and Kristensen, 1989).

### 2.2 Snails rearing

The snails were breed in the VITROME laboratory in IRD Dakar. The temperature and the hygrometry of the breeding room were set at 25–27 °C and between 70 and 80% of humidity, respectively. Before starting the rearing procedure, each snail individual was once assessed for *S. haematobium* and *S. mansoni* cercariae shedding. Each snail was placed in glass tube containing 5 ml of filtered water and exposed to electric light for 30–40 min (Lewis, 2001; Lewis et al., 1986; Senghor et al., 2015). Then after, each tube containing a snail was checked visually for the presence of cercariae. Only negative individuals were conserved in the water from the collection site until they lay eggs. After the eggs hatch, the juveniles specimens, visible to the naked eye, were collected and reared in well water, distilled water, commercial mineral water and ground pump water. The well water and the ground pump water were issued from an urban area in Dakar. The distilled water was produced in the laboratory of VITROME-IRD and the mineral water was purchased commercially.

For each snail species, four batches of 25 juveniles individuals were isolated in plastic container filled with 500 ml of distilled water, commercial mineral, ground pump water and well water, representing therefore four breeding conditions. Each breeding condition was supplemented with 0.15 g of dried lettuce leaves and renewed as necessary and the water was changed weekly. In each container, the pH, conductivity and temperature of the water were measured daily. In addition, the concentration of Cl, NH_4_, and NO2 was measured for each species, the growth of the individuals was evaluated weekly in each type of water by measuring the size of the shell using a digital microscope. The distance between the apex and the spinal notch was considered to measure the size of *B. senegalensis* and *B. truncatus* specimens. The diameter of the shell was used to measure the size of *B. pfeifferi.* The mortality of each species was evaluated in a daily basis. An individual was considered dead if shrinking in its shell, unable to climb in the wall of the container, to hang on to the dried lettuce leaves.

### 2.3 Snails resistance to salinity

*B. truncatus*, *B. senegalensis* and *B. pfeifferi* resistance to salinity was evaluated. Well water was used to prepare three saline solutions at 1.5 g/l, 3.5 g/l and 4 g/l of sodium chloride. For each species, 20 juveniles aged of 3 days were breed in each saline solution and in well water as control. For each condition, breeding container was filled with 500 ml of the adequate saline or control solution and supplemented with 0.15 g of dried lettuce leaves and renewed as necessary. The mortality rate and the growth were monitored daily and each five day, respectively. The procedure for monitoring the growth of snails is identical to that described in section 2.2 “snails rearing”.

### 2.4 Data analysis

The survival of each species according to the different type of water was compared by log-rank (Mantel-Cox) test in the Kaplan Meier analysis and the hazard ratio was given. Bonferroni multiple comparisons test was used to make comparison among multiple groups if the variance by two-way Anova was statically significant. P < 0.05 (two-tailed) was considered to indicate a statistically significant difference.

## 3 Results

### 3.1 The physico-chemical quality of snail breeding water

The mean pH was 6.16, 6.44; 7.26 and 7.28 in distilled, commercial mineral water, well water and ground pump water, respectively. The mean conductivity measured was 24.09 μS/cm, 233.06 μS/cm, 1083 μS/cm and 1194.48 μS/cm in distilled water, commercial mineral water, well water and ground pump water, respectively. The concentration of Cl, NH_4_, NO_2_ or each type of water is indicated in Table 1. Overall, the concentration of chloride ions was greater in well water (119.955 mg/l) and ground pump water (78.475 mg/l) compared to commercial mineral water (20.63 mg/l) and distilled water (1.43 mg/l). The concentration of ammonium was almost the same in well water (0.175 mg/l), ground pump water (0.27 mg/l), commercial mineral water (0.235 mg/l) and distilled water (0.26 mg/l). In ground pump water and well water the contraction of nitrite was at 1.34 mg/l and 0.875 mg/l, respectively. It was found at trace levels in commercial mineral water (0.005 mg/l) and absent in distilled water.

**Table 1:** Chloride, ammonium and nitrite ions concentration (mg/l) in each type of water.

	Ground pump water	Well water	Mineral water	Distilled water
Cl	78.475	119.955	20.63	1.43
NH_4_	0.27	0.175	0.235	0.26
NO_2_	1.34	8.75	0.005	0.000

### 3.2 Snails survival rate (SR) according the different type of water

#### 3.2.1 B. pfeifferi

**Figure 1:**
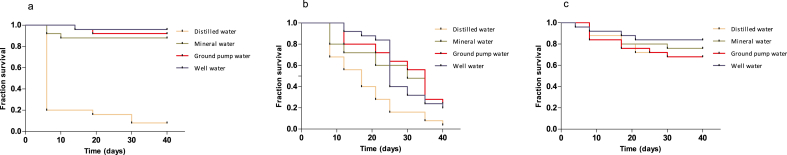
Survival of *B. pfeifferi*, *B. senegalensis* and *B. truncatus* in different water sources; *B. pfeifferi* survival rate was high in commercial mineral water, well water and ground pump water and low in distilled water (log rank X^2^ = 61.94, df = 3, p < 0.0001) (1a). At day 40, low survival rate (24%) of *B. senegalensis* was observedin commercial mineral water and ground pump water. it was at 20% in well water and was very low in distilled water (4%) (log rank X^2^ = 13.24, df = 3, p = 0,004) (1b). High survival rate, between 68% and 84% (log rank X^2^ = 1.86, df = 3, p = 0.60), was observed with *B. truncatus* in all water sources (1c).

High survival rate of *B. pfeifferi* was observed in both commercial mineral water, ground pump water and well water unlike to the high death rate observed in distilled water (log rank X^2^ = 61.94, df = 3, p < 0.0001) (Figure 1a). Despite different trends, there was no significant difference of *B. pfeifferi* survival rate in commercial mineral water, ground pump water and well water (p = 1.162). In well water, the survival fraction was stable at day 14 with survival rate at 96%. In the commercial mineral water and ground pump water the mortality rate was stable at day 10 and 19 respectively, with a survival rate at 88% and 92%, respectively. Poor survival rate was observed in distilled water with an early loss (day 5) of 80% of *B. pfeifferi* population and at day 30, the surviving fraction was stable at 8% until the end of the follow up at day 40. The median survival time was 6 days in distilled water and exceeded 50% in the well, ground and mineral water. Compared to well water, the hazard ration (HR) was 31.76 (95% confidence interval CI = 11.27 to 89.49; p < 0.0001), 2.09 (CI = 0.4040 to 20.84; p = 0.28) and 1.966 (CI = 0.2013 to 19.19; p = 0.56) in distilled water, commercial mineral water and ground pump water, respectively.

At day 40, the mean size of *B. pfeifferi* individuals was at 5.84, 6.95, 7.30 and 7.43 mm in commercial mineral water, distilled water, ground pump water and well water, respectively (Table 2)*.* However, there was no significant difference (P > 0.05) according to Bonferroni multiple comparisons test.

**Table 2:** Snail final mean size (mm) at the end of the follow up in different water sources.

	Ground pump water	Well water	Mineral water	Distilled water
*B. truncatus*	7,158	7,638	6,219	6,234
*B. pfeifferi*	7,303	7,438	5,84	6,95
*B. senegalensis*	8,742	8,827	8,507	6,206

For each species, the size of snails is larger with well water and pump water compared to distilled and commercial mineral water.

#### 3.2.2 B. senegalensis

At day 40, *B. senegalensis* survival rate was 24% in commercial mineral water and ground pump water, 20% in well water and was very low (4%) in distilled water (log rank X^2^ = 13.24, df = 3, p = 0,004). In contrast to the observations with *B. pfeifferi*, the behavior of *B. senegalensis* is marked by a constant decrease of it population despite that it was stable during the ten first days in all type of water (Figure 1b). The median survival time was 35, 30, 25 and 17 days in ground pump water, commercial mineral water, well water and distilled water, respectively. The HR was at 3.38 (CI = 0.1276 to 1.232; p = 0.001), 0.952 (CI = 0.4709 to 1.926; p = 0.89) and 0.769 (CI = 0.3852 to 1.646; p = 0.53) in distilled water, commercial mineral water and ground pump water, respectively, when compared to well water. The mean size of individuals was 6.20 mm, 8.5 mm, 8.74 mm and 8.82 mm in distilled water, commercial mineral water, ground pump water and well water, respectively (Table 2) (P > 0.05, Bonferroni multiple comparisons test).

#### 3.2.3 B. truncatus

High survival rate, between 68% and 84% with no significant difference between the different type of water was observed for *B. truncatus* (log rank X^2^ = 1.86, df = 3, p = 0.60). At day 40, the surviving fraction of *B. truncatus* was at 68% in ground pump water and distilled water when highest survivingl fraction was observed in commercial mineral water (76%) and well water (84%) (Figure 1c). In both commercial mineral water, distilled water and ground pump water, the survival rate was stable at day 30 and at day 20 in well water. The HR was at 1,541(CI = 0.4329 to 5.488; p = 0.50), 2.084 (CI = 0.6544 to 6.637; p = 0.21), and 2.099(CI = 0.6581 to 6.695; p = 0.21) with commercial mineral water, distilled water and ground pump water, respectively, compared to well water.

The mean size observed was 6.21 mm, 6.23 mm, 7.15 mm and 7.63 mm in commercial mineral water, distilled water, ground pump water and well water, respectively (P > 0.05, Bonferroni multiple comparisons test) (Table 2).

### 3.3 Adaptation of snails to salinity

#### 3.3.1 B. pfeifferi

**Figure 2:**
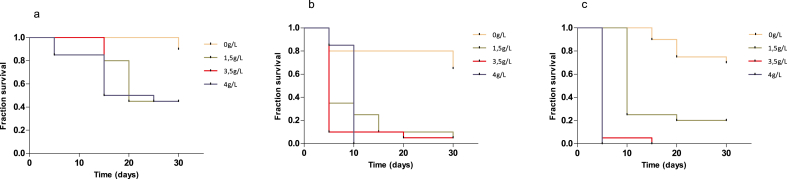
Survival of *B. pfeifferi*, *B. senegalensis* and *B. truncatus* in increasing saline solutions; 45% of *B. pfeifferi* individuals survived to both three saline solutions (log rank X^2^ = 9.86, df = 3, p = 0.0198) (2a). *B. senegalensis* survival rate was low (5%) at day 30 in both 1.5 g/l and 3.5 g/l solution but population collapsed at day 10 in 4 g/l (2b). *B. truncatus* population reached 100% mortality at day 5 and 15 in 4 g/l and 3.5 g/l solution, respectively and persisted with a survival rate at 20% in 1.5 g/l (2c).

*B. pfeifferi* displayed a high tolerance to salinity with a survival rate at 45% at day 30 in both three saline concentration (1.5, 3 and 4 g/l) compared to 90% of survival rate observed in well water (log rank X^2^ = 9.86, df = 3, p = 0.0198) (Figure 2a). The median survival time was 20 days in the three saline solutions. Compared to the well water, the HR was 0.149 (CI = 0.045 to 0.484, p = 0.001), 0.1391 (CI = 0.04084 to 0.4737, p = 0.001) 0.1486 (CI = 0.04600 to 0.4798) in 1.5, 3.5 and 4 g/l saline solution, respectively. At day 30, the mean size of *B. pfeifferi* was at 6.079, 5.956, 5.624 and 5.24 in 0 g/l, 1.5 g/l, 3.5 g/l and 4 g/l saline solutions, respectively (Table 3).

**Table 3:** Snail final mean size (mm) at the end of the follow up in well water (0 g/l) and in saline well water.

	0 g/l	1.5 g/l	3.5 g/l	4 g/l
*B. truncatus*	4,699	4,138	1.742	1.325
*B. pfeifferi*	6,079	5,965	5,624	5,24
*B. senegalensis*	8,876	4,922	1,890	1,870

Snail size for each specie was larger in well water compared to saline solution. The size of snail decreased with increasing salinity. The population reached 100% mortality before the end of the follow up.

#### 3.3.2 B. senegalensis

A low tolerance to saline water was observed with *B. senegalensis* despite the maintenance of the population until day 30 in 1.5 and 3.5 g/l saline solutions with a survival rate at 5%. However, the population of *B. senegalensis* collapsed at day 10 in high saline solution (4 g/l). In the 0 g/l, the survival rate was at 65% at day 30 (p = 0.0002) (Figure 2b). The median survival time was 5 days in 1.5 and 3.5 g/l saline solutions and 10 days in 4 g/l. The HR was 0,095 (CI = 0.03476 to 0.2649, p < 0.0001), 0.062 (CI = 0.01932 to 0.2026, p < 0.0001) and 0.116 (CI = 0.04155 to 0.3276, p < 0.0001) in 1.5, 3.5 and 4 g/l saline solutions compared to well water. At day 30, their mean size was at 6.079 mm, 5.956 mm, 5.624 mm and 5.24 mm in 0 g/l, 1.5 g/l, 3.5 g/l and 4 g/l saline solutions, respectively (Table 3).

#### 3.3.3 B. truncatus

At day 30, the survival rate was at 70% and 20% in well water and in 1.5 g/l saline solution while it collapsed at day 5 and day 15 in 4 g/l and 3.5 g/l saline solution (log rank X^2^ = 33.34, df = 3, p < 0,0001) Figure 2c. The median survival time was at 10 days in 1.5 g/l saline solution and at 5 days in 4 g/l and 3.5 g/l saline solutions. The HR was at 0.131 (CI = 0.045 to 0.377, p = 0.0002), 0.020(CI = 0.006 to 0.069, p < 0.0001) and 0.020(CI = 0.005 to 0.068, p < 0.0001) in 1.5, 3.5 and 4 g/l saline solutions compared to well water. At day 30 the mean size observed was at 4.69, 4.138 in well water and 1.5 g/l saline solution. In 3.5 and 4 g/l saline solutions, the mean size was 1.742 mm and 1.325 mm at day 15 and day 5, respectively ((Table 3).

## 4 Discussion

In this study, we investigated the development and survival of snails, intermediates hosts of *Schistosoma spp*, under laboratory conditions using four types of water and increasing saline solutions. In our knowledge, this is one of the rare studies assessing the behavior of snails vectors under different type of water tto establish a laboratory colony. This study revealed that *B. pfeifferi*, *B. truncatus* and *B. senegalensis* behaved differently to well water, ground pump water, commercial mineral water and distilled water. *B. pfeifferi* was able to grow in all type of water except in distilled water. At the same time *B. truncatus* development was not hampered by any type of water. *B. senegalensis* showed a delicate adaptation to all type of water particularly to distilled water. These results highlight that snails species may have different adaptation to laboratory breeding conditions or at least to the water used for this purpose. In this study, it is revealed that, the survival trend of *B. pfeifferi* and *B. truncatus* was almost identical using well water, ground pump water and commercial mineral water. Distilled water was only favorable for *B. truncatus* survival and it gave the same survival trend as compared to well water, ground pump water and commercial mineral water. The relatively poor survival rates observed with *B. senegalensis* compared to *B. pfeifferi* and *B. truncatus,* confirms the difficulty to maintain this species in laboratory (Wright, 1959). However, it was possible to keep alive during 40 days approximately 30 % of the population of *B. senegalensis* in commercial mineral water, well water and ground pump water. This study also highlighted the ability to maintain different species of snails in a laboratory in Sub-Sahara Africa where bilharzia burden represents a significant health issue. This could contribute considerably to the capacity building of bilharzia control programs and particularly to the training of malacologists. It could also facilitate the establishment of experimental research protocols on parasitic/snail interactions or test various anti-snail strategies.

The observations made with well water, ground pump water seem to be consistent with previous studies using the fresh water from the snails' natural habitat for their rearing in laboratory (Kalinda et al., 2017; Kariuki et al., 2017; Mcclelland, 1964; Najarian, 1961; Swartz et al., 2015). However, the use of fresh water from snail habitat and ground pump water may be constraining if snails have to be kept in the laboratory for a long period of time. Using natural fresh water, whatever its origin, needs to be stored permanently or to have a systematic supplying system. In addition, storage of fresh water would increase the risk of microorganism development that could hamper snail growth or interfere with laboratory experiments, hence the need to heat it sometimes before use (Kariuki et al., 2017). In another hand, the use of natural fresh water may pose the problem of reproducibility if the environmental conditions from water collection site undergo major changes. To avoid these inconveniences, tap water could be used for snails breeding in laboratory. Tap water has the advantage of being accessible, however, it may be challenging to use in experimental procedure due to its quality that is susceptible to changes due to chemical treatment or to the presence of metallic ions (Mcclelland, 1964). In this study, no significant difference in the development of the three species of snails was observed in commercial mineral water compared to well or ground pump water. Furthermore, the size comparison did not show any difference when the snail are breed in mineral water or well and ground pump water, suggesting that commercial mineral water can be used to breed snail in laboratory. The use of commercial mineral water provides a standard rearing medium with a known specific composition, unlike well or ground pump water and more generally water from the snails' natural habitat. In addition, it is more accessible and poses fewer long-term storage problems compared to water from the natural habitat. Therefore commercial mineral water could represent a valuable alternative to fresh water used for snails rearing. However, the variability in water composition between different manufacturers should be taken into account. In this study, the ammonium, nitrite and chloride ions were found at variable concentrations in all types of water In addition according to the manufacturer, calcium, bicarbonate and sulfate ions were present in the commercial mineral water with a relatively low conductivity compared to well water and ground pump water. These essential mineral compounds also found in snail breeding sites may have different impact on their development and abundance depending on snail species (Bakhoum et al., 2019; Yacoubi et al., 2007). Their absence or low level concentration in distilled water could explain the high and earlier mortality of *B. pfeifferi* and *B. senegalensis* observed in the distilled water, unlike what is observed with *B. truncatus*. Some studies revealed that this snail species could breed in natural water with medium level of conductivity (Meierbrook et al., 1987) and a pH between 6-7 (Doumenge et al., 1987). It is also necessary to take into account the financial cost of using commercial mineral water which is more expensive than conventional water sources. Therefore, adjusting pH, conductivity, or inclusion of certain chemicals may help to improve water quality and to reach the same standard as commercial mineral water.

Nevertheless, the use of commercial mineral water was able to give same proportion of survival rate as that observed in well water and ground pump water. However, despite that no significant difference in snail size was observed between different type of water, it appears that the snails breed in commercial mineral water were relatively smallest than in well water or ground pump water. But snails were found infested naturally or experimentally with the same size as in our study during which no special conditions were used (Kariuki et al., 2017; Mulero et al., 2019; Mutuku et al., 2014; Senghor et al., 2015; Tchuenté et al., 1999). For example, we did not oxygenate the tanks or to provide additional mineral ions which could lower the mortality rate and increase specimen size (Rodrigues et al., 2020). An in-depth analysis of the quality of the different waters used coupled with an intentional variation of their physico-chemical parameters would allow to develop suitable snails breeding medium.

In this study, we found that the snail species behaved differently to saline environment with *B. pfeifferi* showing a greater resilience compared to *B. truncatus* and *B. senegalensis*. *B. pfeifferi* was described to develop in waters with high level of salinity in natural conditions (Jordan and Webbe, 1982). However, the construction of a dam on SRB has contributed to the retention of fresh water which has favored the development of *B. pfeifferi* and consequently the emergence of intestinal bilharzia in the area (Southgate, 1997; Talla et al., 1990). On the other hand, the low tolerance of *B. senegalensis* to salinity observed in this study seems to corroborate the observations made in Niakhar, Senegal. Indeed, depending on their distance from the ocean, some freshwater sources became salty due to the upwelling of sea water, which considerably reduced the distribution range of *B. senegalensis* (personal observation). We have also found that *B. truncatus* showed increased sensitivity to salinity compared to *B. senegalensis* and *B. pfeifferi,* which could confirm the sensitivity of this species to relatively high levels of conductivity. This could indicate that in nature, this snail could be very sensitive to the saline environment which could limit its development. Moreover, in the delta of the SRB, the population density of this species is higher in areas with lower salinity (Bakhoum et al., 2019). On the other hand, such laboratory research could help to better understand snail population dynamics and preferred habitat, predict their behavior to global environmental change (Yacoubi et al., 2007) and develop control measures such as those attempting to control cercariae through water chlorination (Braun et al., 2020).

## 5 Conclusion

The adaptation of snails to laboratory conditions may depend on the quality of the water used. The use of commercial mineral water with a specific chemical composition associated specific nutrients could help to standardize the conditions of snails breeding. These results could help to better understand the snail behavior in natural conditions. Further studies, evaluating more vital parameters of the different species of snail, should provide a better understanding of their behavior in the laboratory.

## Declarations

### Author contribution statement

Fatou Thiam: Performed the experiments; Analyzed and interpreted the data; Wrote the paper.

Cheikh Bintou Fall, Papa M Gaye, Kokou Abotsi: Performed the experiments.

Bruno Senghor, Arfang Diamanka, Philippe Parola, Babacar Faye: Contributed reagents, materials, analysis tools or data.

Amélé N Wotodjo: Analyzed and interpreted the data.

Cheikh Sokhna: Conceived and designed the experiments.

Doudou Sow: Conceived and designed the experiments; Contributed reagents, materials, analysis tools or data; Wrote the paper.

Souleymane Doucouré: Conceived and designed the experiments; Analyzed and interpreted the data; Contributed reagents, materials, analysis tools or data; Wrote the paper.

### Funding statement

This work was supported by the IRD, Programme Jeunes Equipes Associéés à l'IRD (JEAI).

### Data availability statement

Data will be made available on request.

### Declaration of interests statement

The authors declare no conflict of interest.

### Additional information

No additional information is available for this paper.
